# Influences of relative deprivation on health inequality of rural residents in China: A moderated mediation model

**DOI:** 10.3389/fpsyg.2022.1082081

**Published:** 2022-12-19

**Authors:** ChunHai Tao, Jun Xu, RuoYan Tao, ZiYu Wang, JiaYi Li

**Affiliations:** ^1^School of Statistics, Jiangxi University of Finance and Economics, Nanchang, China; ^2^School of Liberal Arts, Macau University of Science and Technology, Macao, Macao SAR, China

**Keywords:** rural residents, relative income deprivation, health inequalities, psychological capital, mediating effect

## Abstract

Analyzing the relationship between individual relative deprivation and rural residents’ health inequality is a deepening of the research on the social impact of individual relative deprivation. On the theoretical basis of the absolute and relative income hypothesis, using the data of China Family Panel Studies in 2018, taking other residents in the village as the reference group, this paper selects the relative income deprivation and absolute income to comprehensively quantify the generalized impact of farmers’ income gap, introduces the psychological capital guidance mechanism, and constructs a moderated-mediation model for the impact of relative deprivation on the health inequality of rural residents in China from the three dimensions of self-assessment of health, mental health and physical health. The estimation results of the multi-layer model show that the relative income deprivation of rural residents has a significant impact on health inequality, among which the impact of mental health is the strongest. Compared with physical health, the mediated transmission of psychological capital has a more significant impact between the relative deprivation of rural residents and mental health. Compared with low-income groups, high-income residents can better alleviate the negative effect of income relative deprivation on psychological capital poverty and health inequality, and the regulatory effect on physical health is most significant. Therefore, China can improve the health of the rural poor through fiscal policies such as improving the tax system and strengthening the supervision of various government funds.

## Introduction

Since the implementation of China’s reform and opening-up policy, the individual income distribution gap has been widening and the Gini coefficient value has continued to rise (Ren et al., 2021), far exceeding the international warning line. At the same time, the polarization continues to evolve between different economic development regions and income inequality shows significant regional group differences (Lee et al., 2022), with urban income inequality being lower than rural income inequality (Zhao et al., 2021). Widening income disparities can lead to greater relative deprivation (RD) in both economic and social status for relatively disadvantaged groups. Whether in small rural areas, extending to county-level areas or the entire sample in China, income inequality among rural residents increases the RD. This group is economically disadvantaged and has a much higher incidence of poverty than urban residents (Yu and Li, 2021). The surplus rural labor force is gradually moving to the cities, resulting in a thin labor force and poorer production conditions and infrastructure in the areas of origin (Solt, 2020). The majority of the rural labor force is poorly educated and has a limited choice of employment, resulting in slow income growth (Omar and Inaba, 2020). In the long run, the income deprivation among rural Chinese, exacerbated by the vicious circle, has become a social issue of widespread concern among scholars.

Safeguarding farmers’ health is a prerequisite for accelerating rural economic development (Yang and Wang, 2019). In contrast, the healthcare delivery system in rural areas is still under constant development and reform adjustment, with lower accessibility and fewer quality resources (Cai et al., 2021) and a weaker environment still in need of improvement (Kozhimannil and Henning-Smith, 2021). In contrast, the new rural cooperative medical insurance introduced in China has led to changes in the budget constraints of rural households, influencing the economic resources, living arrangements, and care patterns of rural residents (Wittich et al., 2019), which has had some positive effects on their health status, but is far from adequately meeting the high level of demand for quality healthcare resources in rural areas (Cheng et al., 2018). In addition to further improving healthcare conditions in rural areas, strengthening the social security system in rural areas, and achieving full coverage of county and rural healthcare institutions (Li et al., 2021), policy options for achieving health poverty alleviation in rural areas could also start with improving income inequality (Sun et al., 2022).

Grossman (1972) was the first to investigate the factors influencing health, building on the human capital first formally proposed by Becker et al. (1964), which became the theoretical basis for later research. Among the many studies on the influencing factors of health inequalities, all show that income deprivation has a huge impact on health (Yang and Liu, 2018; Boen et al., 2020). As the number of researchers in this field continues to grow, a theoretical system comprising three main perspectives has gradually emerged: the absolute income hypothesis, the relative income hypothesis and the income inequality hypothesis. First, it is believed that it is primarily the absolute income gap that inhibits the health status of rural residents. For example, Rodgers (1979) studied the relationship between income distribution and health status using the Gini coefficient to measure income distribution in several countries. Second, it is argued that RD significantly affects the health status of rural residents and the investment in health status (including health care investment) (Homan, 2019; Abedi et al., 2021). The relative income hypothesis suggests that individuals with lower income levels than others will experience greater psychological stress and burden on their lives, and their health status will be affected (Wildman, 2021). Life expectancy increases with national income *per capita* and the most important factor influencing life expectancy may be the relative distribution of income. Thirdly, it is argued that the health status and behavior of rural residents is influenced not only by the income status of their own households, but also by the income status of other villagers in the same village (Harada and Sumi, 2020). The lower the ranking of annual *per capita* household income of rural residents’ households, the lower the probability of positive cognition on their self-health status (including mental health and physical health) and actual health consumption (Chokshi, 2018); the RD effect of rural residents on their health behavior also varies depending on the absolute difference in *per capita* household income (Fan et al., 2020).

The direct impact of relative income deprivation on the health status of rural residents is now more commonly studied within the academic community (Fang and Saks, 2021), while the indirect impact mechanisms are less discussed and less well documented (Maykrantz et al., 2021). The introduction of psychological capital, a positive state of mind, has provided direction for influence mechanism research (Darvishmotevali and Ali, 2020) and has important implications for socio-economic development (Stratman and Youssef-Morgan, 2019). Related research has concluded that reducing income disparities can affect the psychological capital of the related group, and that psychological capital also has an impact on health levels (Morgan et al., 2019). So could psychological capital be an essential factor and pathway for relative income deprivation to the health levels of rural residents?

The study attempts to review, analyze and evaluate the relevant literature on the impact of RD on health inequalities, explore the existing problems, clarify the direction of development and guide the implementation of health policies for rural residents. Based on this, the RD index is selected for empirical analysis and the selection of the reference group is introduced; furthermore, the theoretical models of the relative income hypothesis and the absolute income hypothesis are applied to construct a hypothesis model to empirically test the moderating effect of average annual household income and the mediating effect of psychological capital, so as to clarify the path of RD affecting the health status of rural residents in China, with a view to enriching the existing relevant research perspectives. This study aims to provide references and empirical evidence for deepening the reform of the income distribution system in rural areas.

## Hypothesis development

The impact of RD on health inequalities among rural residents may be the result of direct and indirect effects (Nesson and Robinson, 2019). First of all, RD may have a direct impact on health inequalities among rural residents. The specific analysis is as follows: First, as the annual *per capita* income of households decreases, the level of attention and demand for health consumption upgrades such as home care, health care, culture and entertainment is bound to decrease (Costa-Font et al., 2021), Compared to daily consumption products such as electrical appliances and household goods, clothing and accessories, rural residents may not value the usefulness of nutrition services such as health care, thus underestimating health care and reducing investment in higher-level needs for nutrition and health (Nie et al., 2021), including reducing the total investment and share of health care consumption.

Second, a strong sense of RD can trigger individuals to adopt deinstitutionalized or negative coping styles (Srivastava et al., 2021), specifically in the case of rural people’s labor production, where they may feel relatively deprived for not receiving a commensurate quality return for the same amount of time spent working (Gu et al., 2019). As rational human beings, if a significant relative deprivation is felt over a long period of time, ‘corrective’ or ‘compensatory’ action will be taken. Although the time spent in productive labor may increase due to the reverse transmission (Song and Smith, 2019), however, as income distribution mechanisms are still inadequate at this stage, it is difficult for farmers who feel significant relative deprivation to have the same resource endowment to compete rationally to improve their situation and balance their psychological (Zhao, 2019). Ultimately, the income gap is widened due to the Matthew effect between individual farmers, which to a certain extent discourages monitoring and investment in self-health management; on the other hand, problems such as mental or physical health gaps may not be detected in time, and can only be treated passively when they become serious, thus repeatedly widening the health gap (Bittlingmayer et al., 2021), this has resulted in a generally low level of utilization, efficiency of access, and depth of coverage of rural healthcare.

Third, as the positive externalities of improved productive living conditions do not compensate for the health status of rural residents in the short term, the higher the RD level, the higher the opportunity cost for rural residents to adopt health management (Song et al., 2019), and the more likely they are to lack the motivation to improve their health and health literacy, and to choose the healthier lifestyle and medical services that suit them based on their temporary income level and social status (Cheesmond et al., 2019). In summary, the RD’s direct effect on health inequalities among rural residents is likely to manifest itself as a positive amplifying utility. Based on this, the following research hypothesis is proposed:

*H1*: Relative deprivation will directly affects health inequalities among rural residents, and this effect manifests itself as a significant contribution.

Afterwards, RD may indirectly affect farmers’ health inequality by influencing residents’ psychological capital characteristics, which are essentially farmers’ longitudinal RD feelings with reference to their past selves or future expectations. For the same absolute amount of income, rural residents with lower RD levels are more convenient and better positioned to maintain their positive, healthy and sunny psychological profiles (Yang and Liu, 2018), they may also prefer to pursue the higher levels of need for ‘respect’ and ‘self-fulfilment’. It is therefore an important objective of the reform of China’s income distribution system to ensure a basic and stable income source for the residents, which not only reduces their worries about medical treatment, but also reduces their dependence on the government and the community, reduces their psychological stress and increases their self-esteem, and makes them more optimistic about their health (Xiong et al., 2021). Existing studies generally agree that relative income levels can have an important impact on health status through psychological mechanisms, and that an increase in the income distribution gap brings more feelings of loss and frustration to low-income groups (Richman et al., 2019), and that stress from life triggers psychological imbalance, generating negative emotions such as anxiety, pessimism, dissatisfaction and negative attitudes toward life, which eventually lead to the development of bad habits such as smoking and drug use, and in the long run, the health of low-income residents will deteriorate.

As RD increases, farmers may compress the range of social activities to reduce spending on higher-level needs, thereby increasing disconnection from society and inside-out group comparison anxiety (Isaacs et al., 2018). However, residents who reduce the health care consumption level such as health care, fertility, nutrition and medical treatment generally have low expectations on the investment benefits of health care. They may not have the power to invest in health expenditure or continue to consume more advanced and scientific nutrition and health lifestyles. Even if the consumption of medical and health expenditure is increased unconsciously, the stability of health management may still be relatively low (Peng et al., 2019).

Although these studies are helpful to understand the RD mechanism affecting the health inequality of rural residents, there are two shortcomings: first, they overestimate the RD’s impact on physical health. The research shows that, due to the gradual improvement of basic medical security for rural residents in China and the substantial reduction of basic medical service costs, the impact was not as large as expected, caused by the RD impelling farmers to maintain physical health and treat non-psychological diseases (Li et al., 2018); Second, it ignores the pathways by which RD affects mental health by influencing the mood of rural residents’ lives and their future expectations. As RD increases, rural residents may reduce the variety of social activities without changing the size of household health expenditures (Jalil et al., 2022), and a reduction in the variety of social activities (i.e., suppressing the demand for higher levels of health) may reduce the optimality of the allocation of health factors in rural areas (Yang L. et al., 2019), thus potentially widening health disparities among residents. Based on this, this paper argues that RD may indirectly affect health inequalities among rural residents by affecting psychological capital, and proposes the following hypothesis:

*H2*: Relative deprivation significantly and negatively affects the psychological capital of rural residents.

*H3*: Psychological capital significantly and negatively affects the health inequalities of rural residents.

*H4*: Relative deprivation can indirectly and positively correlate with health inequalities among rural residents by affecting their psychological capital.

The absolute income hypothesis theory is an important perspective for understanding the psychological decision-making and behavior of relative income deprivation (Mackenbach, 2019), focusing on the differences in decision-making and behavior of households in different bands of absolute income (Li et al., 2022; Wang et al., 2022; Zhou et al., 2023). However, little attention has been paid to the moderating role of different bands of absolute *per capita* household income in the RD’s impact on health inequalities among rural residents (Lyu and Sun, 2020). Overall, rural households in different ranges of household income differ in terms of demographics, employment preferences, and capital accumulation, and may show variability in health care investment when RD increases (Hastings, 2019; Yang X. Y. et al., 2019). At the same time, total household income *per capita* is closely related to the socio-economic behavior of rural households in terms of labor supply, household living environment and consumption and savings, and therefore has a significant impact on rural households’ healthiness decisions and resource allocation (Kordan et al., 2019), which may lead to adjustments in the proportion and focus of rural households’ inputs on health factors.

Further, in terms of absolute household income, rural households above the average level generally spend less money and time on basic livelihoods and tend to focus on higher-level needs satisfaction (Zeng and Wei, 2021), with less adjustment to basic livelihoods inputs, and thus RD may have less impact on health inequalities among rural residents at this stage. For rural households below the average level, with the increase of the proportion of the expenditure on basic living security, the effective labor force of rural households is expected to deteriorate in the future, and the family burden is gradually increasing (Sesen and Ertan, 2019). Rural residents may regard maximizing the immediate benefits of basic life as the main goal of income distribution, ignoring the development of psychological dynamics, which reinforces the inequality gap in health status. To sum up, when in different stages of absolute family income, RD may have different effects on the health inequality of rural residents.

Based on this, this paper proposes the following hypothesis:

*H5*: The impact of relative deprivation on the health inequality of rural residents is related to the absolute family income.

*H6*: The impact of relative deprivation on the psychological capital of rural residents is related to the absolute family income.

**Figure 1:**
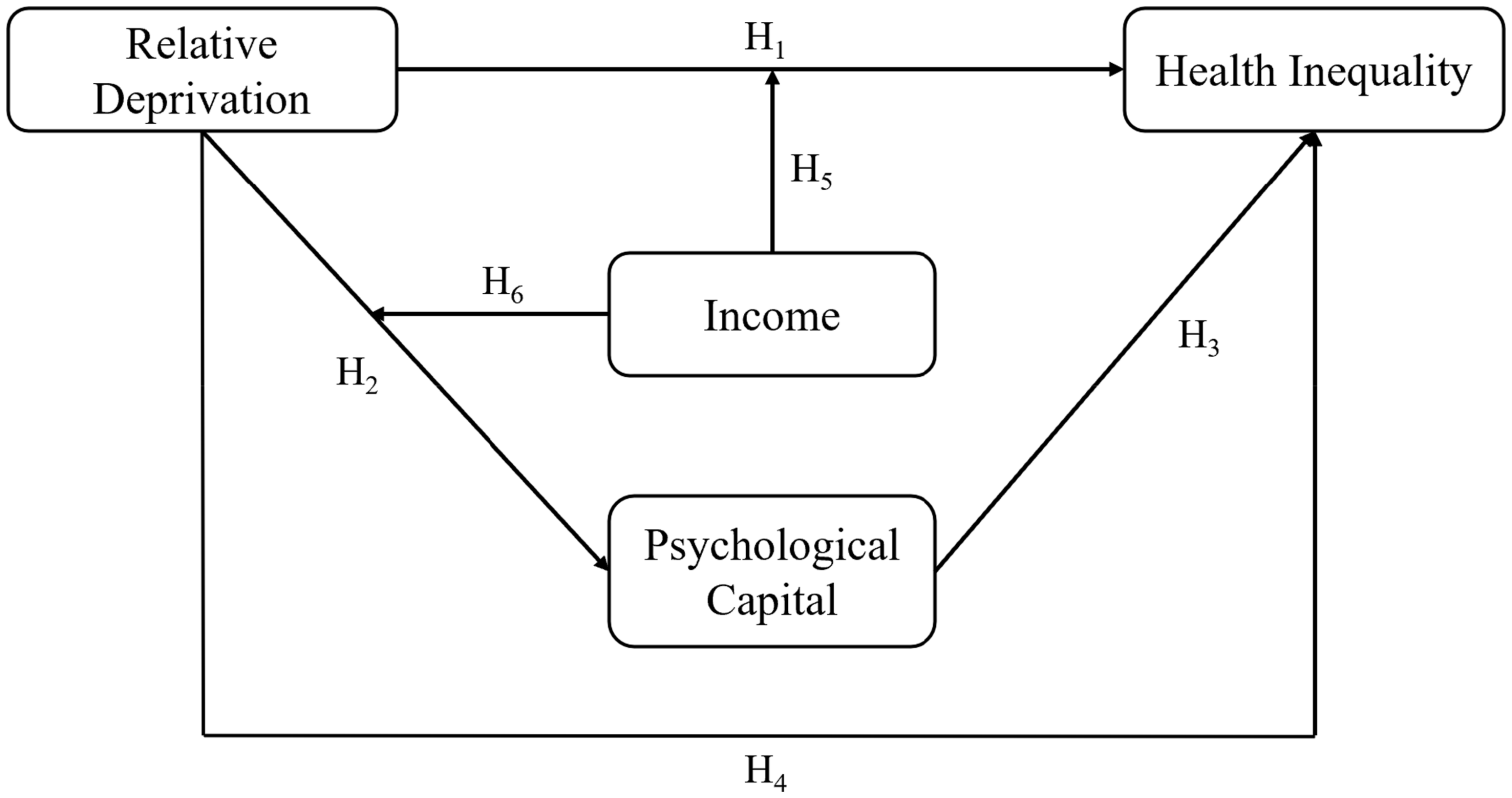
Theoretical model.

In summary, the following research model can be formed according to each research hypothesis (as shown in Figure 1).

## Data sources, description of variables, and measurement models

### Data sources

The data used in this article are from the 2018 China Family Panel Studies (CFPS). Organized by the Institute of Social Science Survey (ISSS) of Peking University, China, the survey aims to reflect social, economic, demographic, educational and health changes in China by tracking and collecting data at the individual, household and community levels, and to provide a data base for academic research and public policy analysis.

The CFPS sample covers 25 provinces/municipalities/autonomous regions in China, with a target sample size of 16,000 households, and includes all household members in the sample. All baseline household members and their future blood/adopted children, as defined by the 2010 baseline survey, are genetic members of the CFPS and are permanently tracked, with four main types of questionnaires—community, household, adult and child - and six rounds of data collected in the China Big Survey have been published to date.

To reflect the latest situation, this paper uses data from the 2018 CFPS to analyze the impact of relative deprivation on health inequalities among rural residents in China. The sample of residents in rural areas was retained, and those with missing data on important variables were removed, resulting in a sample size of 17,721 for analysis. Also, to ensure consistency of magnitude, the main continuous variables are standardized in this paper, and all data processing and empirical analysis is carried out in Stata 17.0 software. The average age of rural residents in the sample was 45.96 years old, 49.7% were male, 80.7% were married, the average household size was 4.53 persons, and the average annual household income was 16,437 yuan. The education level of the sample was generally moderate, with 20.3% illiterate or semi-literate people and 25.8% with high school education or above; the employment rate of the sample was high, with 81.8% of rural residents being employed; the participation rate of the sample in medical insurance was 85.9%, and most of them participated in China’s New Rural Cooperative Medical Scheme (“NRCMS”), which has achieved a high level of coverage in rural areas.

### Description of variables

(1) Relative income deprivation (RID). The geographical ties in rural areas are stronger. The geographical scope, mainly focusing on villages, is concerned with the social life and communication of rural residents, and their perception of relative income is also mainly compared with villagers in the same village. Therefore, this paper uses the village as the reference unit to measure the relative deprivation of individual rural residents. According to relative deprivation theory, within a cluster, if the income level of residents is lower, the income disadvantage is greater and the level of income inequality is higher, which means the degree of income relative deprivation suffered is higher.

According to Kakwani (1980) definition of the relative deprivation index (RD for short), assuming that x represents a reference group with a sample size of n, the income vector ( $x_{1},x_{2},\ldots ,x_{i},x_{n}$ )is obtained by sorting the income of farmers in the village from smallest to largest, where $x_{1}\leq x_{2}\ldots \leq x_{i}\leq x_{n}$ . Thus the RD index for the i th resident $x_{i}$ compared to the *j*th resident, relative deprivation index $RD\left(x_{j}-x_{i}\right)$ for the i th resident expressed as:


(1) $$RD\left(x_{j}-x_{i}\right)=\{\begin{array}{c} x_{j}-x_{i}\;if\;x_{j}>x_{i}\\ 0\;if\;x_{j}\leq x_{i} \end{array}$$

On the basis of Equation 1, the $RD\left(x_{j}-x_{i}\right)$ suffered by the i th resident $x_{i}$ can be expressed in three forms as follows:

① The Yitzhaki Index. Yitzhaki (1979) was a pioneer in the study of RD measures, stating that individual deprivation arises from a comparison with those in the reference group who earn more than them, and is calculated as:


(2) $$Yitzhaki=\frac{1}{n}\overset{n}{\underset{k=i+1}{\sum }}\left(x_{k}-x_{i}\right)=\gamma_{x_{i}}^{+}\left(\mu_{x_{i}}^{+}-x_{i}\right)$$

② The Deaton Index. Deaton Index builds on the Yitzhaki Index by taking into account the average income of the members of the reference group in order to satisfy the requirement of size invariance (Deaton, 2003), which is calculated as:


(3) $$Deaton=\frac{1}{n\mu_{x}}\overset{n}{\underset{k=i+1}{\sum }}\left(x_{k}-x_{i}\right)=\gamma_{x_{i}}^{+}\left[\frac{\mu_{x_{i}}^{+}-x_{i}}{\mu_{x}}\right]$$

③ The Podder’s index. In their studies, some scholars have assumed that income follows a normal distribution (Eibner and Evans, 2005), replacing income in the Yitzhaki index with income in logarithmic form, a logarithmic form of the deprivation index first proposed by Podder (1996), which is calculated as:


(4) $$Podder=\frac{1}{n}\overset{n}{\underset{k=i+1}{\sum }}\left(\ln x_{k}-\ln x_{i}\right)=\gamma_{x_{i}}^{+}\left[\mu_{\ln x_{i}}^{+}-\ln x_{i}\right]$$

The mean income of the reference group with income over $x_{i}$ is expressed as $\mu_{x_{i}}^{+}$ , $\gamma_{x_{i}}^{+}$ is the percentage of the total sample whose income exceeds $x_{i}$ , and $\mu_{x}$ is the mean income of the total sample，since the income of each individual farmer is not identical and their position on the income distribution varies (Benzeval and Judge, 2001; Deaton, 2003), the Deaton index can reflect different RD levels of residents.

④ Income quartiles. Drawing on Li and Zhu (2008), the income percentile ranking of each individual household in the village was calculated for each sample i . If the individual i ‘s annual *per capita* household income was the highest in the village, the income percentile ranking was taken as 100; if the individual i ‘s annual *per capita* household income was the lowest in the village, the income percentile ranking was taken as 0.

In summary, the Deaton Index and income quartiles, which are the most frequently used and formalized in relevant studies, were selected to measure objective RD among rural residents.

(2) Health inequalities. CFPS has more detailed questions on individual health status, and this paper selects self-rated health, mental health and physical health, which have been most widely used in the literature, to measure individual farmers’ health inequalities.

① Self-rated health. Self-rated health as a measure of individual health, although highly subjective, is highly correlated with objective health indicators such as mortality (Mcewen et al., 2009), and is a comprehensive positive health indicator that is widely used, as well as being the primary dependent variable in this paper.

② Mental health. CFPS questionnaire includes a measure of mental health, Center for Epi-demiologic Studies Depression (also known as the CES-D scale), to measure residents’ mental depression. In this paper, the mean of the CES depression score was calculated as a measure of mental health.

③ Physical health. In addition to the above indicators, this paper also takes reference from Li et al. (2014) and selects Physical Activity of Daily Living (PADL) limitation indicators to measure physical health of residents. This section of the CFPS survey was interviewed for the age group >45 years and this paper will construct a physical health (PADL) indicator for each activity restricted, with higher scores being associated with less restricted activity and better physical health function.

(3) Psychological capital. Psychological capital refers to the psychological state of rural residents in their productive lives, including psychological feelings about their current lives, their hopes for their future lives and their optimism when they encounter difficulties. The study chose the indicator of rural residents’ confidence in their future to characterize their psychological capital.

(4) Income inequality. Considering the relevance of the absolute and relative income hypothesis theories, in addition to measuring relative income deprivation from each dimensional deprivation indicator, this paper also selects the absolute income measure of annual *per capita* household income to portray the RD’s impact on health inequality.

(5) Control variables. At the individual level this paper selects variables such as age, gender, marital status, work status, years of education and health insurance, at the household level the variables of annual household income *per capita*, household drinking water and household size, and at the social level the variables of neighborhood trust, stranger trust and human relations.

Table 1 shows the information on the descriptive statistical analysis of all the variables:

**Table 1:** Descriptive statistical analysis.

Variable type	Variable name	Variable meaning	Mean	SD	Min	Max
Explanatory variable	Podder	RD measured using village as a reference group geographical extent (−)	8.38	1.10	0.46	12.88
	Deaton	RD measured using village as a reference group geographical extent (−)	0.40	0.26	0	1
	Percent_inc	Percentage of *per capita* annual household income in the village by village as a reference group (+)	0.50	0 0.29	0	1
Explained variable	Self-rated Health	Very unhealthy = 1, relatively unhealthy = 2, average = 3, relatively healthy = 4, very healthy = 5	2.97	1.31	1	5
	CESD	The Center for Epidemiological Studies-Depression(−)	13.80	4.11	8	32
	PADL	Physical Activities of daily living(+)	6.62	1.09	0	7
Moderating variable	Average Family income	Family income/ Family size (logarithmic)	9.28	0.93	5.01	13.85
Mediating variable	Psychological Capital	The level of confidence in your future	4.15	0.99	1	5
Individual control variable	Age	Natural age of respondents (in years)	45.96	19.64	9	100
	Gender	Female = 0; male = 1	0.50	0.50	0	1
	Marital status	Separation, divorce, widowhood, never married = 0; married, cohabitation = 1	0.80	0.40	0	1
	Work	Employed = 1, others = 0	0.82	0.39	0	1
	Education	Illiterate / semi-illiterate / not in school = 0, primary school = 1, junior high school = 2, high school / technical secondary school / vocational high school = 3, junior college = 4, bachelor’s degree = 5, master = 6	1.53	1.29	0	6
	Insurance	Enroll in Medicare = 1, None = 0	0.92	0.26	0	1
Family control variable	metotal	Total medical expenditure (logarithmic)	4.86	3.45	0	12.89
	Water	Other = 0; Tap water = 1	0.62	0.48	0	1
	num_Child	Children number	4.53	2.11	1	21
Social control variable	Neighbors	The level of trust in the neighbors(1–10)	6.79	2.20	0	10
	Strangers	The level of trust in strangers(1–10)	2.12	2.21	0	10
	Interperson	Relationship quality (1–10)	7.14	2.05	0	10

### Measurement models

#### Baseline model

The multi-layered model in this paper consists of two layers, individual and village, where the first layer is individual and the second layer is village level. The basic model is set up as follows:


(5) $$Health\;Inequality_{ic}=\beta_{0}+\beta_{1}RD_{ic}+\beta_{2}X_{ic}+\zeta_{c}+\varepsilon_{ic}$$

In Equation 5, health inequality is the health status of residents in village c; RD is the relative deprivation of individual i in terms of income; and *X* is a control variable. Among the explanatory variables studied in this paper, self-rated health status is a dichotomous variable, while mental health and physical health take values as integer variables between 8 and 32, and between 0 and 7. Therefore, this paper uses a dichotomous logistic model and a multilayer linear regression model to estimate the RD’s effect on health inequalities among rural residents, respectively, with the former converting coefficient values into incidence odds for impact analysis.


(6) $$Odds=\frac{p\left(srh_{ic}=1|x_{ic},\zeta_{c}\right)}{1-p\left(srh_{ic}=1|x_{ic},\zeta_{c}\right)}$$

In this Equation 6, $srh_{ic}$ is the outcome of self-rated health status of individual i in village c; $p\left(srh_{ic}=1|x_{ic},\zeta_{c}\right)$ is the conditional probability of self-rated health status being ‘bad’ and $1-p\left(srh_{ic}=1|x_{ic},\zeta_{c}\right)$ is the conditional probability of self-rated health status being ‘good’. The incidence is the ratio of the probability of a rural resident’s self-assessed health status being ‘bad’ to the probability of a self-assessed health status being ‘good’.

#### Mediating effects model

RD may act on farmers’ health inequalities through the intermediate transmission of psychological capital. Drawing on Baron et al.’s research, a stepwise regression method was used to construct a model mediated by psychological capital, as follows:


(7) $$HI_{i}=\alpha_{0}+\alpha_{1}RD+\sum \alpha_{2}X_{i}+v_{1}$$

(8) $$PC_{i}=\beta_{0}+\beta_{1}RD+\sum \beta_{2}X_{i}+v_{2}$$

(9) $$HI_{i}=\chi_{0}+\chi_{1}RD+\chi_{2}PC+\sum \chi_{3}X_{i}+v_{3}$$

In Equations 7–9, HI is health inequality, PC is psychological capital, $\alpha_{1}$ denotes the total effect of relative deprivation on health inequality, $\beta_{1}$ denotes the effect of relative deprivation on psychological capital, and the coefficient $\chi_{2}$ denotes the direct effect of psychological capital on health inequality. Substituting Equation 8 into Equation 9 gives a further mediating effect $\beta_{1}\chi_{2}$ of the intermediate transmission mechanism. It should be emphasized that self-rated health is a ‘0–1’ variable, so the logit model regression. is used to regress the equation with it as the dependent variable.

#### Moderating effects model

To test the moderating effect of absolute annual household income *per capita* on relative deprivation and health inequality, an interaction term between relative deprivation and annual household income *per capita* was introduced and modeled as follows:


(10) $$HI_{i}=\gamma_{0}+\chi_{1}RD_{i}+\gamma_{2}AFI_{i}+\sum \gamma_{3}X_{i}+v_{4}$$

(11) $$HI_{i}=\gamma_{0}+\chi_{1}RD_{i}+\gamma_{2}AFI_{i}+\eta RD_{i}{}^{*}AFI_{i}+\sum \gamma_{3}X_{i}+v_{4}$$

(12) $$PC_{i}=\varphi_{0}+\chi_{1}RD_{i}+\varphi_{2}AFI_{i}+\sum \varphi_{3}X_{i}+v_{5}$$

(13) $$PC_{i}=\varphi_{0}+\chi_{1}RD_{i}+\varphi_{2}AFI_{i}+\mu RD_{i}{}^{*}AFI_{i}+\sum \varphi_{3}X_{i}+v_{5}$$

In Equations 10–13, AFI represents annual *per capita* household income, RD × AFI is the interaction term between relative deprivation and annual *per capita* household income, and *X* is a control variable.

## Main results and discussion

### Baseline analysis

Equations 1–3 in Table 2 report the estimated results of multi-layer logistic model regarding the effect of relative deprivation on the self-rated health status of rural residents (OR values are reported). Equation 1 shows that the RD’s OR, as measured by the Deaton Index, is 0.531 less than 1 and is statistically significant at the 1% level, indicating that an increase in the ranking of rural residents in terms of relative deprivation in their village significantly reduces the log-incidence of their self-rated health status as ‘good’. Equation 2 is an estimated effect of RD, as measured by the Podder Index, on the self-rated health status of rural residents, with an OR of 0.907 less than 1 and statistically significant at the 1% level, with the same practical significance as Equation 1. Equation 3 shows the estimated results of the impact of the percentile ranking of the *per capita* annual income of rural households in the village on their self-assessment health status. The OR value is significantly greater than 1, indicating that the logarithm incidence of the “good” self-assessment health status of rural people is significantly increased by 1.842% for every 1% increase in the percentile ranking of the *per capita* annual income of rural households in the village.

**Table 2:** Results of multi-layer model estimation of the RD’s effect on the health status of rural residents.

Variable name	Model 1	Model 2	Model 3	Model 4	Model 5	Model 6	Model 7	Model 8	Model 9
	Self-rated health			CES-D			PADL		
Deaton	0.531^***^			0.788^***^			−0.258^**^		
	(0.068)			(0.196)			(0.111)		
Podder		0.907^***^			0.292^***^			−0.163^***^	
		(0.028)			(0.071)			(0.044)	
Percent _inc			1.842^***^			−0.824^***^			0.416^***^
			(0.213)			(0.174)			(0.101)
age	0.959^***^	0.961^***^	0.957^***^	0.028^***^	0.028^***^	0.027^***^	−0.012^***^	−0.012^***^	−0.012^***^
	(0.003)	(0.003)	(0.003)	(0.005)	(0.005)	(0.005)	(0.004)	(0.004)	(0.004)
gender	1.288^***^	1.268^***^	1.325^***^	−0.387^***^	−0.361^***^	−0.374^***^	−0.049	−0.054	−0.052
	(0.087)	(0.087)	(0.088)	(0.101)	(0.097)	(0.098)	(0.061)	(0.060)	(0.061)
marri	0.969	0.977	0.961	−0.972^***^	−0.917^***^	−0.894^***^	0.176^**^	0.141^*^	0.154^*^
	(0.088)	(0.089)	(0.086)	(0.128)	(0.122)	(0.123)	(0.087)	(0.084)	(0.086)
work	1.305^***^	1.351^***^	1.235^**^	−0.226^*^	−0.267^**^	−0.244^*^	0.728^***^	0.744^***^	0.755^***^
	(0.111)	(0.117)	(0.103)	(0.134)	(0.128)	(0.129)	(0.078)	(0.076)	(0.077)
Education	1.124^***^	1.146^***^	1.107^***^	−0.189^***^	−0.168^***^	−0.162^***^	0.044^**^	0.037^**^	0.031
	(0.021)	(0.022)	(0.021)	(0.029)	(0.029)	(0.029)	(0.019)	(0.019)	(0.019)
insurance	1.001	1.019	0.949	−0.445^**^	−0.507^***^	−0.503^***^	−0.080	−0.080	−0.093
	(0.118)	(0.121)	(0.110)	(0.174)	(0.166)	(0.167)	(0.107)	(0.104)	(0.105)
water	1.031	1.057	1.025	−0.130	−0.121	−0.121	0.075	0.073	0.090
	(0.069)	(0.071)	(0.067)	(0.100)	(0.097)	(0.097)	(0.060)	(0.059)	(0.060)
num_child	1.162^***^	1.151^***^	1.162^***^	−0.253^***^	−0.230^***^	−0.228^***^	0.008	0.002	0.005
	(0.049)	(0.049)	(0.048)	(0.073)	(0.071)	(0.072)	(0.026)	(0.026)	(0.026)
metotal	1.000^***^	1.000^***^	1.000^***^	0.000^***^	0.000^***^	0.000^***^	−0.000^**^	−0.000^**^	−0.000^**^
	(0.000)	(0.000)	(0.000)	(0.000)	(0.000)	(0.000)	(0.000)	(0.000)	(0.000)
Neighbors	1.085^***^	1.082^***^	1.081^***^	−0.233^***^	−0.236^***^	−0.228^***^	0.007	0.005	0.004
	(0.017)	(0.017)	(0.016)	(0.024)	(0.023)	(0.023)	(0.013)	(0.013)	(0.013)
Strangers	1.044^***^	1.042^***^	1.042^***^	−0.032	−0.033	−0.037^*^	0.013	0.012	0.014
	(0.016)	(0.017)	(0.016)	(0.023)	(0.022)	(0.022)	(0.013)	(0.013)	(0.013)
Interperson	1.056^***^	1.058^***^	1.061^***^	−0.188^***^	−0.185^***^	−0.190^***^	0.008	0.010	0.011
	(0.017)	(0.017)	(0.017)	(0.025)	(0.024)	(0.024)	(0.014)	(0.014)	(0.014)
Pseudo R2	0.124	0.119	0.122	0.081	0.078	0.078	0.123	0.126	0.129
N	6,212	5,892	6,585	6,198	6,633	6,571	1,492	1,543	1,518
Log likelihood	−3,036	−2,922	−3,175	−16,999	−18,169	−17,992	−2,268	−2,342	−2,311

*p < 0.05; **p < 0.01; ***p < 0.001.

Table 2, Equations 4–6, report the estimated results of the multilevel linear regression model regarding RD on the mental health of rural residents. The coefficient estimates can be directly interpreted as the change in CES-D scores reflecting mental health due to changes in the independent variables. The effect of the RD, as measured by both the Deaton Index and the Podder Index, was significant with a positive coefficient, while it was equally significant with a negative coefficient on the effect of the income percentile ranking variable of the annual *per capita* income of rural residents in their village, suggesting that relative deprivation has a negative effect on the mental health of residents in rural areas. Specifically, for every 10% increase in Deaton index, rural residents’ CES-D scores reflecting mental health will increase by 0.0788 points; For every 0.1 increase in the Deaton index, the CES-D score of rural middle-aged and elderly people reflecting mental health increased by 0.0292 points; The CES-D score of rural households decreased by 0.824 points for each increase in the *per capita* annual income percentile ranking of the village.

Table 2, Equations 7–9, report the estimated results of the multilevel linear regression model regarding RD on the physiological health of rural residents. The coefficient estimates can be directly interpreted as changes in PADL scores reflecting physiological health resulting from changes in the independent variables. The effects of the RD, as measured by the Deaton and Podder indices, are both significant with negative coefficients, while the effects of the income percentile ranking variable of the annual *per capita* income of rural households in their village are equally significant with positive coefficients. This suggests that RD has a negative impact on the physical health of rural residents. Specifically, for every 10% increase in the Deaton Index, rural residents’ PADL scores reflecting physical health decreased by 0.0258 points; for every 0.1 increase in the Deaton Index, rural people’s PADL scores reflecting mental health increased by 0.0163 points. The PADL scores of rural residents decreased by 0.416 points for every one place increase in the income percentile ranking of their village. Both self-rated health and mental health reflect respondents’ subjective assessment of their own health status, suggesting that relative income disparities act directly on individuals’ health status in the form of psychological stress.

### Mediated effects analysis

This study first used stepwise regression to test mediating effects. First, the main effect of RD on health inequalities was tested, and as shown above, the main effect of RD on health inequalities was statistically significant; Second, individual-level variables, family-level variables and social-level variables were used as control variables, and the regression equation was established with RD as the independent variable and psychological capital as the dependent variable. The results showed that the negative predictive effect of RD on psychological capital was statistically significant ( β = −0.192, *p* < 0.01). Third, controlling for individual-level, family-level and society-level variables, the independent variable RD and the mediating variable psychological capital were simultaneously placed in the regression equation, with health inequality as the dependent variable.

The results showed that the positive predictive effect of psychological capital on self-rated health was statistically significant ( β = 0.336, *p* < 0.01, *R*^2^ = 0.102), and the negative predictive effect of RD on self-rated health increased ( β = − 0.928, *p* < 0.01, *R*^2^ = 0.116).Thus, psychological capital partially mediated the effect of RD on self-rated health; the negative predictive effect of psychological capital on mental health was statistically significant ( β = − 0.996, *p* < 0.01, *R*^2^ = 0.128), and the positive predictive effect of RD on mental health was enhanced ( β = 1.405, *p* < 0.01, *R*^2^ = 0.127).Thus, psychological capital partially mediated the effect of RD on mental health; the positive predictive effect of psychological capital on physiology was statistically significant ( β = 0.092, *p* < 0.01, *R*^2^ = 0.091), and the negative predictive effect of RD on physiological health was enhanced ( β = − 0.407, *p* < 0.01, *R*^2^ = 0.124).Therefore, psychological capital partially mediates the effect of RD on physical health; thus, hypothesis 2, hypothesis 3, and hypothesis 4 are supported.

The results of further Bootstrap analysis (Table 3) showed that the 95% confidence intervals for the direct and indirect effects of psychological capital in the process of income deprivation on the self-rated health were −0.231 to −0.169 and −0.019 to 0.009, respectively, with confidence intervals not containing 0, indicating that both the direct and indirect effects were both significant, which suggests that psychological capital partially mediates the process by which it influences self-rated health. Similarly there is a partial mediating effect of psychological capital between mental health and physical health. The mediating effects of self-rated health, mental health and physical health accounted for 6.7%, 11.7%, and 4.2%, respectively. In summary, the mediating effect of psychological capital on mental health is more significant than that of self-rated health and physical health.

**Table 3:** Analysis on the mediating effect played by psychological capital.

Explanatory variable	Effect type	Effect value	Standard error	95% CI	Effect proportion
Psychological Capital to self-rated health	Direct effect	−0.200	0.015	[−0.231, −0.169]	93.3%
	Indirect effect	−0.014	0.003	[−0.019, −0.009]	6.7%
Psychological Capital to CESD	Direct effect	1.405	0.035	[0.117, 0.256]	88.3%
	Indirect effect	0.186	0.138	[1.135, 1.675]	11.7%
Psychological Capital to PADL	Direct effect	−0.018	0.005	[−0.027, −0.008]	95.8%
	Indirect effect	−0.407	0.044	[−0.493, −0.322]	4.2%

### Moderating effects analysis

This study used hierarchical regression to test the moderating effect of the absolute income amount on direct effect. The steps are as follows: ① health inequalities (categorized as self-rated health, mental health and physical health) are used as the dependent variables and control variables (individual control variables, family control variables and social control variables) are placed into the regression equation. ② put the independent variable RD into the regression equation; ③ put the moderating variable mean annual household income into the regression equation; ④ put the interaction term of the centralized RD and average family income(AFI) into the regression equation.

As shown in Table 4, the interaction terms for RD and absolute income were statistically significant predictors of psychological capital (Model 10: β = −0.169, *p* < 0.01, *R*^2^ = 0.096). This suggests that absolute income moderates the relationship between RD and psychological capital and has a negative effect on the original effect, which means higher absolute income of rural residents reduces the negative effect of RD on psychological capital, and Hypothesis 5 was verified. Similarly testing the moderating effect of absolute income on the total effect (as shown in Table 4), the interaction term between RD and absolute income was statistically significant in predicting self-rated health, physical health (Model 11: β = − 0.732, *p* < 0.01, *R*^2^ = 0.103; Model 13: β = 0.427, *p* < 0.01, *R*^2^ = 0.115), and not mental health.This suggests that AFI has a moderating effect on the relationship between RD and self-rated health and physical health, both of which are negative, while the moderating effect on mental health is not significant, which means higher absolute income of rural residents reduces the negative effect of RD on self-rated health and physical health, and Hypothesis 6 were partially verified.

**Table 4:** Regulated mediating effect test.

Variable	Model 10	Model 11	Model 12	Model 13
	PC	SRH	CESD	PADL
Podder	−0.169^***^	−0.732^***^	−0.262^***^	0.427^**^
	[−0.243, −0.094]	[−0.952, −0.512]	[−0.449, −0.076]	[0.025, 0.828]
AFI	8.46e-07^*^	8.60e-06^***^	−0.265^**^	0.621^***^
	[−1.48e-07, 1.84e-06]	[3.83e-06, −1.34e-05]	[−0.319, −0.212]	[0.495, 0.748]
Podder * AFI	0.235^***^	2.35e-05^***^	−0.061	−0.301^***^
	[0.171, 0.298]	[−1.08e-05, −3.63E-05]	[−0.165, 0.043]	[−0.517, −0.084]

*p < 0.05; **p < 0.01; ***p < 0.001. PC indicates psychological capital, SRH indicates self-rated health, CESD indicates The Center for Epidemiological Studies-Depression, PADL indicates physical activities of daily living, and AFI indicates average family income.

## Research discussion

### Conclusion

This study introduces psychological capital variables to investigate the relationship between relative income deprivation and health inequalities among rural residents. Based on two theories of income inequality, the absolute income hypothesis and the relative income hypothesis, the study comprehensively examines the association between income deprivation and health inequality under various forms, and further reveals the moderating effect of absolute income amount and the psychological capital-mediated transmission pathway, with the following findings:

First, relative income deprivation among rural residents can be significantly associated with health inequalities, with the strongest association being for mental health. In line with Nesson and Robinson’s (2019) argument for the association between income inequality and health inequality, the findings are similar to those of Richman et al. Thus, improving the country’s urban and rural income distribution system can reduce the income gap between residents. A fairer and more scientific income distribution and redistribution policy can be formulated to reduce the income inequality between urban and rural areas as well as within them as much as possible, thus improving the overall health of the residents. At the same time, due to the uneven development between regions in China, there is a significant income gap and some low-income groups still bear high medical costs, so it is necessary to increase financial investment in healthcare so that low-income groups can also enjoy basic medical treatment.

Second, the effect of psychological capital mediated transmission was more significant between relative deprivation and mental health of rural residents compared to physical health. The findings are consistent with existing research, confirming the significant relationship between relative deprivation, psychological capital and health inequalities (Darvishmotevali and Ali, 2020; Maykrantz et al., 2021). A higher perception of individual income relative deprivation is not conducive to promoting the accumulation of psychological capital and the health status of the population, which stems from a psychological state in which individuals perceive that their benefits are being appropriated and taken away from them with reference to others or other groups, and this psychological stimulus leads to psychosocial imbalances. The higher the perception of poverty, the more negative the psychological impact, creating a certain hatred of the rich, which in turn generates negative emotions and thus affects their health status. If properly guided so that individuals can accept the existence of fair income deprivation, this will not only increase cohesion and identity among residents, but will also improve social integration and promote the physical and mental health of individuals. It also enhances interpersonal cohesion and communication, as well as social status, which can contribute to improving one’s social capital and one’s own health status.

Third, higher income resident groups can better mitigate the reinforcing effects of relative income deprivation on psychological capital deprivation and health inequalities compared to lower income groups, where the moderating effect on physical health is more significant. The findings are consistent with those of Lyu and Sun (2020). Since the absolute income of rural residents has a significant contribution to health status, on the one hand, we can increase the added value of agricultural products by increasing the income sources of rural residents, providing advanced technical support and working conditions for agricultural production, gradually moving down labor-intensive industries to create more labor jobs, reducing the cost of increasing income for rural residents, broadening the marketing channels of agricultural products, and extending the industrial chain of agricultural products. On the other hand, active measures are also being taken to promote the high-quality transfer of surplus rural labor, to increase the income levels of rural residents and to narrow the income gap between them and urban residents.

### Theoretical implications

The research in this paper has some theoretical implications for the introduction of psychological variables into economic sociological research. Most of the existing studies only describe the health status of rural residents from a single dimension, and lack of systematic theoretical analysis (Galloway and Henry, 2014; Li et al., 2014; Guo et al., 2022). Based on the combination of theory and empirical evidence, this paper designs a psychological mediation model that can explore the heterogeneity of relative deprivation on different dimensions of health from the introduction of psychological capital as a psychological perspective, further enhancing the feasibility and applicability of the theoretical model of health behavior in the field of psychobehavior, and providing some basis for further in-depth research. It will be helpful to enrich the existing relevant research and provide a certain basis for further in-depth study. At the same time, focusing on the moderating effects of income, this paper analyzes the influence of absolute income, relative income and income inequality on the health of rural residents, and excavates the deep-seated factors that affect the health level of rural residents, it fills the blank of this field and is innovative to a certain extent.

### Practical implications

The findings of this paper have certain policy implications. This paper shows that relative deprivation based on income has a negative impact on the health status of rural residents, and that relative deprivation due to income inequality further exacerbates the health problems associated with rural income disparities. Therefore, deepening the reform of rural income distribution is the most crucial aspect of achieving rural health care. In addition to strengthening the health care and social security systems in rural areas, deepening the reform of the income distribution system in rural areas can also alleviate, to a certain extent, the health problems of rural residents brought about by social development phenomena such as urban–rural regional disparities and population ageing. The study can thus provide some practical basis and effect predictions for measures related to the effects of fiscal policy to improve the health of deprived people in China, such as improving the taxation system, strengthening the supervision of various government funds, and realizing tax relief policies for some people with financial difficulties or special circumstances.

### Limitations

This paper has some limitations. Firstly, it is limited to the original data of the research sample, and only a single indicator is selected to measure psychological capital. Although it can explain psychological capital to a certain extent, it has not yet reached the ideal state of multi-dimensional measurement, which may have some impact on the accuracy of the results. The paper also uses only psychological capital variables as mediating variables and does not analyze the mediating role of other factors such as social capital, which should be further verified by structural equation modeling in the future. In addition, this paper does not analyze in depth the effect mechanism of relative income deprivation on the health of different types of residents. Further heterogeneity analysis on different genders, different age stages, and different geographical areas will provide more scientific and precise evidence to support the research on this topic in the future.

## Data availability statement

The original contributions presented in the study are included in the article/supplementary material, further inquiries can be directed to the corresponding authors.

## Author contributions

CT contributed to the model building as well as the article writing. JX and RT cooperated with all of CT’s work and contributed to the empirical analysis and text writing. ZW and JL contributed to the overall quality of literature organization and article revision. All authors contributed to the article and approved the submitted version.

## Funding

This project was supported by the National Social Science Fund of China Project: Statistical Measurement and Process Monitoring of the Upgrading of China's Large Health Industry under the New Pattern of “Dual Circulation” (Grant No.21&ZD150) and Research on the Regional Economic Growth Effect of the Agglomeration of China’s Pharmaceutical Manufacturing Industry from the Perspective of Resource Mismatch (Grant No.20ATJ003).

## Conflict of interest

The authors declare that the research was conducted in the absence of any commercial or financial relationships that could be construed as a potential conflict of interest.

## Publisher’s note

All claims expressed in this article are solely those of the authors and do not necessarily represent those of their affiliated organizations, or those of the publisher, the editors and the reviewers. Any product that may be evaluated in this article, or claim that may be made by its manufacturer, is not guaranteed or endorsed by the publisher.
